# Acute Kidney Injury Following Intraureteral Indocyanine Green Use During Laparoscopic Endometrioma Surgery. Comment on Scholz et al. First Reported Case of Acute Kidney Injury Following Intraureteral Indocyanine Green Administration During Bilateral Endometrioma Excision. *J. Clin. Med.* 2025, *14*, 8758

**DOI:** 10.3390/jcm15197553

**Published:** 2026-09-29

**Authors:** Rebecca Mallick

**Affiliations:** Department of Obstetrics and Gynaecology, University Hospitals Sussex NHS Foundation Trust, Princess Royal Hospital, Lewes Road, West Sussex RH16 4EX, UK; rmallick@doctors.org.uk

We read with great interest the recent case report describing acute kidney injury (AKI) requiring temporary haemodialysis following intraureteral indocyanine green (ICG) instillation during laparoscopic endometrioma excision [1]. The authors should be commended for reporting a serious postoperative complication potentially associated with an increasingly adopted technique for ureteric visualisation.

The report raises an important safety question regarding the off-label intraureteral use of ICG. The unusual clinical course—normal preoperative renal function followed by rapid bilateral anuria, in the absence of identified mechanical obstruction, and a temporary requirement for haemodialysis—appropriately justifies reporting ICG as a suspected contributor. However, as the authors acknowledge, the observation establishes a temporal association rather than a causal relationship. The principal value of the case is therefore hypothesis-generating, prompting closer examination of the possible mechanism and proportionate consideration of its clinical implications.

Postoperative AKI following major pelvic surgery is frequently multifactorial [2]. In the presented case, several recognised contributors were present, including prolonged laparoscopic surgery with pneumoperitoneum, baseline low blood pressure, perioperative blood loss with postoperative anaemia, surgical inflammation, and the physiological stress response associated with complex endometriosis surgery. Each of these factors has independently been associated with perioperative renal dysfunction and may have acted synergistically [2,3]. The patient’s low baseline blood pressure, in particular, may have increased susceptibility to renal hypoperfusion. However, detailed perioperative haemodynamic data, including the duration and severity of any intraoperative hypotension, were not reported, limiting assessment of its contribution. The coexistence of these risk factors makes it particularly difficult to isolate the contribution of intraureteral ICG or establish a direct causal relationship. Other relevant determinants include intraoperative fluid balance and exposure to potentially nephrotoxic medications, although these were not reported in sufficient detail to assess their contribution in this case.

While the temporal relationship between ICG instillation and AKI is notable, a biological mechanism has not been established. Intravenously administered ICG is highly protein-bound, undergoes hepatic uptake and biliary excretion, and is not considered directly nephrotoxic. However, these pharmacokinetic data cannot necessarily be extrapolated to retrograde intraureteral administration, which produces direct urothelial exposure. Theoretical mechanisms might include local urothelial or tubular toxicity, transient obstruction related to ureteric oedema or spasm, or renal injury associated with high-pressure retrograde instillation. These mechanisms remain speculative and have not been substantiated by experimental or epidemiological evidence.

ICG has demonstrated a favourable safety profile across a broad range of surgical and diagnostic applications, including in patients with impaired renal function, although most published evidence relates to intravenous administration. Reports of retrograde intraureteral ICG use have described successful ureteric visualisation with few reported renal complications; however, the available series are small and do not allow the incidence of rare renal adverse events to be reliably estimated [4,5].

Our group has also reported a prospective consecutive series of 50 patients undergoing complex benign gynaecological procedures using intraureteral ICG-guided ureteric visualisation [5]. We did not observe postoperative AKI, anuria, dialysis requirement, or any renal complication attributable to ICG administration. However, our cohort was not designed to assess rare adverse events and may not have included patients with the same combination of baseline and perioperative risk factors as the index case. Therefore, the absence of renal complications in our series does not exclude a rare event or establish safety in higher-risk patients. To date, few renal complications have been reported following intraureteral ICG administration, but the true incidence remains uncertain.

We agree with the authors that continued vigilance and systematic reporting are essential as fluorescence-guided surgery evolves. Intraureteral ICG remains a potentially valuable technique for real-time ureteric identification, but the available evidence is insufficient to define its renal safety profile or determine the balance of benefits and risks. Larger prospective studies and multicentre registries using standardised intraureteral ICG protocols and systematically capturing administration details, perioperative risk factors, and predefined renal outcomes will be required to determine whether the reported event represents a true ICG-related complication or a rare manifestation of multifactorial postoperative AKI.

The case serves as a valuable reminder that renal function should be monitored carefully following complex pelvic surgery. The index case is clinically important and appropriately hypothesis-generating, prompting vigilance and further systematic investigation. However, a single observation cannot confirm causality or establish intraureteral ICG as the mechanism of renal injury in the absence of supporting mechanistic or epidemiological evidence.

## Data Availability

No new data were created or analyzed in this study. Data sharing is not applicable to this article.
